# A pear S1-bZIP transcription factor PpbZIP44 modulates carbohydrate metabolism, amino acid, and flavonoid accumulation in fruits

**DOI:** 10.1093/hr/uhad140

**Published:** 2023-07-21

**Authors:** Hong Wang, Kexin Xu, Xiaogang Li, Bárbara Blanco-Ulate, Qingsong Yang, Gaifang Yao, Yiduo Wei, Jun Wu, Baolong Sheng, Youhong Chang, Cai-Zhong Jiang, Jing Lin

**Affiliations:** College of Horticulture, Nanjing Agricultural University, Nanjing 210014, China; Institute of Pomology, Jiangsu Academy of Agricultural Sciences/Jiangsu Key Laboratory for Horticultural Crop Genetic Improvement, Nanjing 210014, China; College of Horticulture, Nanjing Agricultural University, Nanjing 210014, China; Institute of Pomology, Jiangsu Academy of Agricultural Sciences/Jiangsu Key Laboratory for Horticultural Crop Genetic Improvement, Nanjing 210014, China; Institute of Pomology, Jiangsu Academy of Agricultural Sciences/Jiangsu Key Laboratory for Horticultural Crop Genetic Improvement, Nanjing 210014, China; Department of Plant Sciences, University of California, Davis, Davis, CA 95616, USA; Institute of Pomology, Jiangsu Academy of Agricultural Sciences/Jiangsu Key Laboratory for Horticultural Crop Genetic Improvement, Nanjing 210014, China; School of Food and Biological Engineering, Hefei University of Technology, Hefei 230009, China; Department of Plant Sciences, University of California, Davis, Davis, CA 95616, USA; College of Horticulture, Nanjing Agricultural University, Nanjing 210014, China; Institute of Pomology, Jiangsu Academy of Agricultural Sciences/Jiangsu Key Laboratory for Horticultural Crop Genetic Improvement, Nanjing 210014, China; Institute of Pomology, Jiangsu Academy of Agricultural Sciences/Jiangsu Key Laboratory for Horticultural Crop Genetic Improvement, Nanjing 210014, China; Department of Plant Sciences, University of California, Davis, Davis, CA 95616, USA; Crops Pathology and Genetics Research Unit, United States Department of Agriculture, Agricultural Research Service, Davis, California, 95616, USA; College of Horticulture, Nanjing Agricultural University, Nanjing 210014, China; Institute of Pomology, Jiangsu Academy of Agricultural Sciences/Jiangsu Key Laboratory for Horticultural Crop Genetic Improvement, Nanjing 210014, China

## Abstract

Fruit quality is defined by attributes that give value to a commodity. Flavor, texture, nutrition, and shelf life are key quality traits that ensure market value and consumer acceptance. In pear fruit, soluble sugars, organic acids, amino acids, and total flavonoids contribute to flavor and overall quality. Transcription factors (TFs) regulate the accumulation of these metabolites during development or in response to the environment. Here, we report a novel TF, *PpbZIP44*, as a positive regulator of primary and secondary metabolism in pear fruit. Analysis of the transient overexpression or RNAi-transformed pear fruits and stable transgenic tomato fruits under the control of the fruit-specific E8 promoter demonstrated that *PpZIP44* substantially affected the contents of soluble sugar, organic acids, amino acids, and flavonoids. In *E8::PpbZIP44* tomato fruit, genes involved in carbohydrate metabolism, amino acid, and flavonoids biosynthesis were significantly induced. Furthermore, in *PpbZIP44* overexpression or antisense pear fruits, the expression of genes in the related pathways was significantly impacted. *PpbZIP44* directly interacted with the promoter of *PpSDH9* and *PpProDH1* to induce their expression, thereby depleting sorbitol and proline, decreasing citrate and malate, and enhancing fructose contents. *PpbZIP44* also directly bound to the *PpADT* and *PpF3H* promoters, which led to the carbon flux toward phenylalanine metabolites and enhanced phenylalanine and flavonoid contents. These findings demonstrate that *PpbZIP44* mediates multimetabolism reprogramming by regulating the gene expression related to fruit quality compounds.

## Introduction

Fruit quality influences consumer preference and market competitiveness. Therefore, improving fruit quality has been the cornerstone for breeders and scientists. Fruit quality includes taste, aroma, texture, and nutritional quality, in addition to shelf life and other visual attributes [1]. The content and species of soluble sugars and organic acids are the fundamental compounds for fruit growth, development, and ripening and determine the fruit flavor at the edible stage [2, 3]. Fatty acids, amino acids and vitamins, and secondary metabolites such as phenylpropanoids, flavonoids, and phenolics are vital factors in determining the health-promoting quality of fruits [1, 4, 5]. As with most complex agronomic traits, fruit quality is affected by primary and secondary metabolic shifts [6]. In the Rosaceae species, sorbitol and sucrose are the primary end products of photosynthesis, synthesized in leaves and transported to fruits through the phloem [7]. Then, sucrose is resynthesized via sucrose phosphate synthase (SPS) or converted to fructose and glucose by invertases (INV) or sucrose synthase (SUS) [2, 8, 9]. Sorbitol is either accumulated or converted mainly to fructose by sorbitol dehydrogenase (SDH) in fruits [7]. Unlike soluble sugar metabolites, the organic acids accumulated in the fruit mainly depend on the *de novo* synthesis in fruit cells [3]. Malate, an intermediate product of the tricarboxylic acid (TCA) cycle, is the most abundant organic acid determining flavor quality in pear [3]. Organic acids accumulate in young fruits to generate cellular energy for respiratory and intermediates for the biosynthesis pathway, then gradually decrease and achieve a palatable sugar:acid ratio, which is a significant indicator of fruit quality [3]. Amino acids are derived from different intermediates of glycolysis and the TCA cycle, and aromatic amino acids (AAA) are synthesized from the shikimate pathway [10]. Amino acids are essential for maintaining fruit quality and play positive roles in human health [10]. Flavonoids, as the most abundant phenolic substance in plants, regulate various plant biological processes, including fruit coloration, resistance to UV-B damage and defense against biotic and abiotic stress, and also possess a wide range of physiological features that benefit human health, including radical scavenging, anti-inflammation and anti-aging [11]. Elucidation of the underlying metabolic regulation responsible for changes in fruit attributes is essential for providing scientific guidance for improvement of fruit quality.

Biosynthesis and accumulation of primary and secondary metabolites are regulated by transcription factors (TFs) [12–14]. TFs can activate the collaborative expression of multiple genes, thereby effectively regulating the reprogramming of primary or secondary metabolic pathways [13]. Evidences suggest that one subgroup of the basic leucine zipper (bZIP) transcription factors (S1-bZIP group) mediates sugar-related regulatory gene expression and are required for sugar signaling and plays important roles in the regulation of fruit quality and stress response [15]. Conserved upstream open reading frames (uORFs) in the 5′-untranslated regions of the S1-bZIP group genes encode a sucrose control peptide associated with sucrose-induced translation repression [16]. Genome editing *FvebZIPs1.1* uORF resulted in a diverse amount increase in sugar content in strawberries [17]. Compared with the wild type (WT), overexpression of strawberry *FvbZIP11* in tomatoes significantly increased the total soluble solids and the contents of soluble sugars [18]. Transgenic lines with overexpressing tomato *SlbZIP1* and *SlbZIP2* contained 1.5-fold higher sucrose/glucose/fructose than WT control, and increased levels of several amino acids [19]. These findings suggest that the S1-bZIP TFs have a broad role in sugar accumulation and regulating carbon–nitrogen balance. However, whether or how pear S1-bZIP TFs regulate primary and secondary metabolic shifts in fruit remains unclear.

The discovery and identification of novel TFs are vital for successfully improving fruit quality, especially in woody plants that generate commercially superior fruits with better palatability, nutrient balance, and health benefits [1]. Pear is a popular fruit worldwide. For several years, there has been increased interest in the consumption of pear fruits. Hence, cultivation areas have been increasing, production has intensified, and new cultivars have been developed [20]. The great demand for pear fruit is associated with its taste, texture, and nutritional value. Enhancing fruit quality through metabolite manipulation or breeding has been an essential in increasing Asian pear quality. We have recently developed the ‘Sucui 1’ [*Pyrus pyrifolia* (Burm.f.) Nakai.,] variety by crossing ‘Huasu’ and ‘Cuiguan’. ‘Sucui 1’ fruits have improved quality and extrinsic features and an extremely short growth period, and could be used as an excellent material for studying the mechanism of fruit quality or other agronomy traits in pears. In this study, we identified a critical regulator, a member of the S1-bZIP group *PpbZIP44*, that affected fructose accumulation, organic acids contents, amino acid, and flavonoid levels. Our finding provides new insights into understanding the regulatory mechanism for future metabolic engineering to improve fruit quality.

## Results

### Identification of *PpbZIP44* and its expression patterns during development and ripening in pear fruit

**Figure 1:**
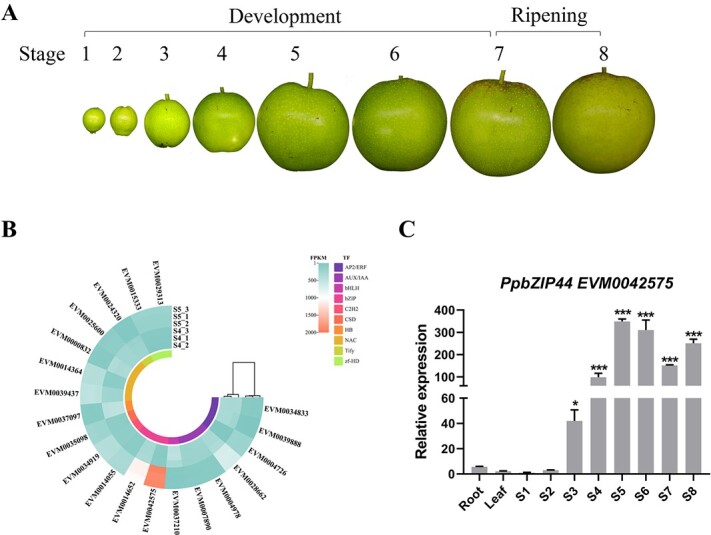
Fruit development stages and transcription abundance of identified genes. A. Eight fruit developmental and ripening stages were selected for analysis in pear. B. Heatmap hierarchical clustering showing top 20 TFs and FPKM value of S1-bZIP members at fruit development stages 4 and 5 based on transcriptome data in ‘Sucui 1’. Colors represented different TFs types. C. Time course expression profiles of *PpbZIP44* in the root, leaf, fruit development, and ripening processes of ‘Sucui 1’ using qRT-PCR. Sarcocarp samples with moving peels measuring 0.4-cm thick were collected every 15 days after full bloom. Three independent biological replicates containing at least five fruits for every experiment were performed for each stage. Expression levels were normalized to the levels of pear *GAPDH*. The asterisks indicated values determined by the Dunnett *t* test to significantly differ from stage 1 (^*^P < 0.05, ^***^P < 0.001).

To gain insight into the structural characteristics of the S1-bZIP TFs, we performed a phylogenic analysis using 105 pear bZIP TFs from the ‘Cuiguan’ pear genome database [21] and their orthologs in *Arabidopsis thaliana*, *Solanum lycopersicum*, *Musa nana, Fragaria ananassa*, *Vitis vinifera*, *Oryza sativa*, and *Malus pumila*. The phylogenetic analysis assigned 11 PpbZIPs, which were noted as red dots to S1-bZIP group (Fig. S1A and Table S2). We also constructed a comprehensive phylogenetic tree using pear S1-bZIP proteins and S1-bZIP TFs from other species (Fig. S1B and Table S2). Pairwise protein sequence alignment found that 3 of 11 PpbZIPs, such as EVM0005411, EVM0009313, and EVM0014055, were identified as orthologs of AtbZIP11, whereas EVM0014652, EVM0000485, and EVM0042575 have the highest homology with AtbZIP44 (Fig. S1B and Table S2). In addition, EVM0041187 and EVM0041928 were identified as orthologs of AtbZIP53. To perform gene expression analysis, we collected samples from leaves, roots, and fruits, including seven developmental stages (15, 30, 45, 60, 75, 90, and 93) and one ripening stage (100 DAP) of ‘Sucui 1’ fruits (Fig. 1A). Our previous RNA-seq data showed that the significantly high transcription abundance of an S1-bZIP gene EVM0042575 (*PpbZIP44*) was observed at stage 5 (S5) among the top 20 highest expressed TFs (Fig. 1B). To further examine the expression dynamics of several candidate S1-bZIP genes, we performed the quantitative real-time polymerase chain reaction (qRT-PCR) analysis. Compared with other genes, *PpbZIP44* (EVM0042575) displayed much higher transcript abundances in fruits (Fig. 1C and Fig. S2). Especially, the expression level of *PpbZIP44* was the highest at S5 and 3.5-fold higher than that at stage 4 (S4). A higher expression level of *PpbZIP44* was also found at stage 6 (S6) and the ripening stage 8 (S8).

### Overexpression or silencing of *PpbZIP44* modulated sugar: Acid ratio in pear fruits

**Figure 2:**
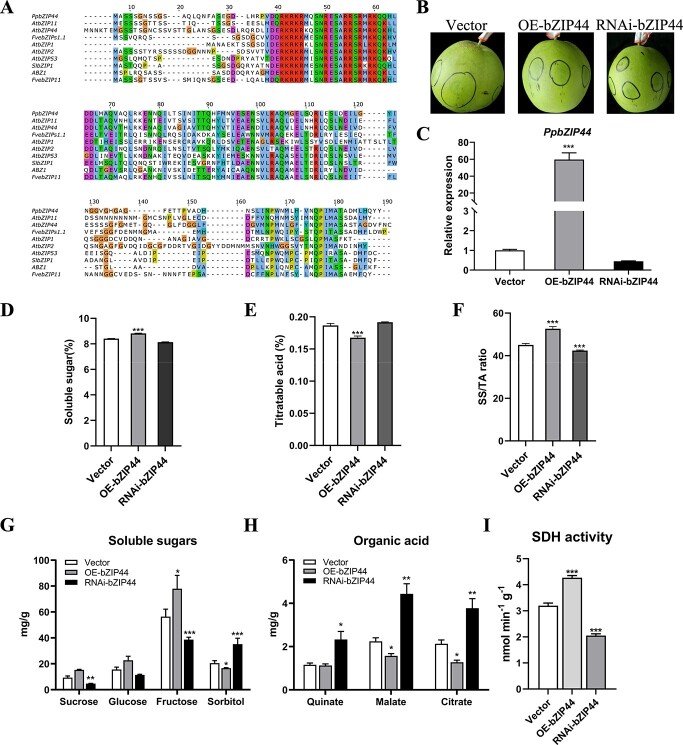
Transient transformation of *PpbZIP44* in pear fruits. A. Multiple protein sequence alignments of *PZIP44* and other S1-bZIP proteins in different species. B. Transient assays in ‘Sucui 1’ pear using overexpression (OE-) or RNAi (RNAi) or empty vector (Vector) of *PpbZIP44*. C. The expression levels of *PpbZIP44* in the fleshy tissue around the infiltration sites of transformed pear fruits using qRT-PCR. The content of total SS (D) and TA (E), SS/TA ratio (F), individual soluble sugars contents (G), individual organic acids contents (H), and SDH activity (I) in transformed pear fruits and control pear fruits. Bars represented the mean value ± SE (n ≥ 3). The asterisks indicated values that were determined by the Dunnett *t* test to significantly differ from their empty vector or WT control (^*^P < 0.05, ^**^P < 0.01, ^***^P < 0.001).

We cloned the *PpbZIP44* gene from the sweet pear variety ‘Sucui 1’ with early maturation. The 474-bp open reading frame (ORF) of *PpbZIP44* encoded a protein of 157 amino acids with one conserved bZIP domain (Fig. 2A). To examine the biological function of *PpbZIP44* in fruit, we first carried out the transient overexpression and silencing assays in pear fruit. Fruits were collected a week before ripening and injected with the transient overexpression-, RNAi-, and empty-vector (Fig. 2B). At 7 days after injection, the expression levels of *PpbZIP44* were 59-fold higher in the OE-bZIP44 and 0.44-fold lower in the RNAi-bZIP44 fruit, when compared with that in the empty vector control (Fig. 2C).

The total soluble sugar displayed a significantly higher level in OE-PpbZIP44 and lower in RNAi-PpbZIP44 than in fruits with the empty vector (Fig. 2D). Similarly, the titratable acid content was significantly reduced in OE-PpbZIP44 but increased in RNAi-PpbZIP44 compared with the control (Fig. 2E). The sugar:acid ratio in OE-bZIP44 was 52.67, higher than 45.08 in WT and 42.43 in RNAi-bZIP44 (Fig. 2F). The fructose and sorbitol contents significantly differed between the transgenic fruits and the empty vector control. Specifically, compared with the empty vector control, the fructose contents increased significantly by 38.17% in OE-bZIP44, whereas they decreased significantly by 31.45% in RNAi-bZIP44. The sorbitol contents decreased by 26.42% in OE-bZIP44, whereas increasing significantly by 72.09% in RNAi-bZIP44. Slight changes in glucose and sucrose contents were observed on the transiently transformed fruits compared with control ones (Fig. 2G). All organic acids contents were significantly increased in RNAi-bZIP44 (Fig. 2H), indicating a role for *PpbZIP44* in the TCA cycle. In agreement, the organic acids, malate, and citrate were decreased by 29.96% and 40.05%, respectively, in OE-bZIP44 compared with the empty vector control.

SDH is the essential enzyme in converting sorbitol to fructose. We evaluated the PpSDH enzymatic activity in transiently transformed fruits and observed that its activity was increased approximately 1.3 times in OE-bZIP44 fruits compared with the empty vector control. In the RNAi-bZIP44, PpSDH enzyme activity was decreased by 0.6 times (Fig. 2I).

### Effects of *PpbZIP44* on amino acid contents in transiently transformed pear fruits

We further analyzed the contents of amino acids in transiently transformed pear fruits with OE-bZIP44 and RNAi-bZIP44 constructs. The total amino acid contents of OE-bZIP44 and RNAi-bZIP44 fruits were 18.9% higher and 8.5% lower than those of fruits with the empty vector. Met and Tyr levels were approximately 2-fold higher in OE-bZIP44 than in the empty vector control. Phe, Asp, and Ala levels were 1.1- to 1.2-fold higher in OE-bZIP44 and lower in RNAi-bZIP44 compared with empty vector fruits. Val, Ser, and Pro levels were lower in OE-bZIP44 but higher in RNAi-bZIP44. The levels of Glu, Ile, and NH3 were similar in the fruits with the empty vector as that of OE-bZIP44 or RNAi-bZIP44 (Table 1).

### Fruit-specific overexpression of *PpbZIP44* resulted in **metabolites reprogramming in tomato fruits**

**Figure 3:**
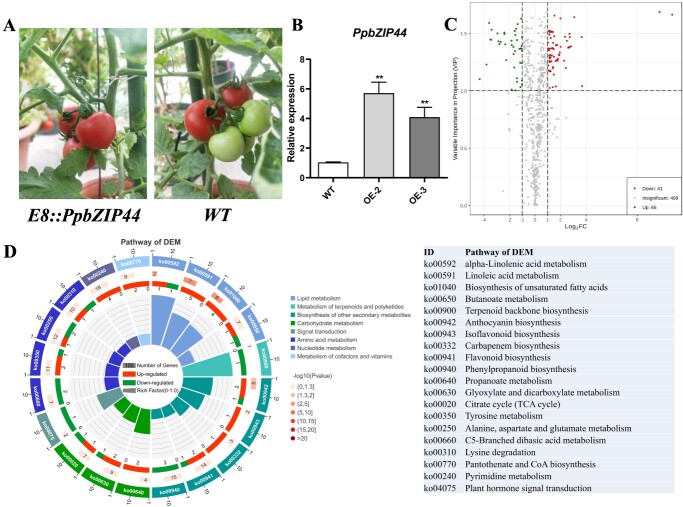
Fruit-specific overexpression of *PpbZIP44* in tomatoes. A. Representative phenotype of *E8::PpbZIP44* tomato fruits. B. The expression levels of *PpbZIP44* in WT and *E8::PpbZIP44* fruits. C. Volcano plot of DEMs in *E8::PpbZIP44* versus WT fruits. D. Summary of top 20 KEGG pathway, significantly overrepresented in DEMs in *E8::PpbZIP44* versus WT fruits. The results are averages ± SE (n ≥ 3) of WT and transgenic lines, each with three technical replicates. The asterisks indicated values determined by the Dunnett *t* test to significantly differ from the WT control (^**^P < 0.01).

To further verify the function of *PpbZIP44*, we attempted to overexpress the *PpbZIP44* in tomatoes due to the significant challenge in pear transformation and unrealistic fruit-forming in wood plants with limited time. The tomato fruit-specific E8 promoter was used to drive *PpbZIP44* expression in tomato fruit. Eight T0 lines were obtained. These lines grew and developed normally (Fig. 3A). The expression of *PpbZIP44* in the transgenic lines was monitored by qRT-PCR. *E8::PpbZIP44–2* (OE-2) and *E8::PpbZIP44–3* (OE-3), which had higher *PpbZIP44* expression in the ripening fruit compared with the WT control (Fig. 3B), were then chosen to obtain a T1 generation for further investigation.

To better characterize the effect of *PpbZIP44* on the contents of compounds, we performed widely targeted metabolomics of *E8::PpbZIP44–2* and WT tomato fruits using multiple reaction monitoring methods (MRM) [22]. We identified 613 metabolic compounds across all samples (Supplementary Table S3). Among these, 108 were classified as differential expression metabolites (DEMs) based on two thresholds [variable importance in projection (VIP) ≥1 and fold-change ≥2 and ≤0.5] when comparing the *E8::PpbZIP44* lines against the WT control (Fig. 3C). Kyoto encyclopedia of genes and genomes (KEGG) enrichment analyses in the DEMs showed that the top overrepresented pathways included lipid metabolism (e.g. biosynthesis of unsaturated fatty acid, linoleic acid, and alpha-linolenic acid metabolism), biosynthesis of other secondary metabolism (e.g. isoflavonoid/flavonoid/flavone and flavonol biosynthesis), amino acid metabolism, and carbohydrate metabolism (e.g. the TCA cycle) (Fig. 3D). Most DEMs in linoleic acid biosynthesis, flavonoid-like biosynthesis, tyrosine metabolism, and alanine, aspartate, and glutamate metabolism were upregulated in the *E8::PpbZIP44* tomato fruits (Fig. 3D).

The changes of the corresponding metabolites in these pathways induced by *PpbZIP44* were further analyzed. The results of widely targeted metabolomics showed that the levels of sucrose and fructose increased >1.1-fold in transgenic fruits compared with WT fruits.

*PpbZIP44* significantly induced the threose level to ~2-fold compared with WT fruits (Supplementary Table S4). The levels of sorbitol, glucose 6-phosphate, fructose 6-Phosphate, fructose-1,6-biphosphate, D-sedoheptuiose 7-phosphate, and 3-phospho-D-glyceric acid were decreased; however, the levels of glucuronic-6,3-lactone, 6-phosphoglucose acid, ribulose-5-phosphate, and phosphoenolpyruvate were >1.5-fold increase in the corresponding transgenic fruits (Supplementary Table S4). Overexpression of *PpbZIP44* decreased the TCA cycle intermediate contents (e.g. fumaric acid, citric acid, malic acid, citraconic acid, 3-Guanidinopropionic acid, α-Ketoglutaric acid) (Supplementary Table S4) and significantly increased the level of succinic acid and amino malonic acid (Supplementary Table S4).

**Figure 4:**
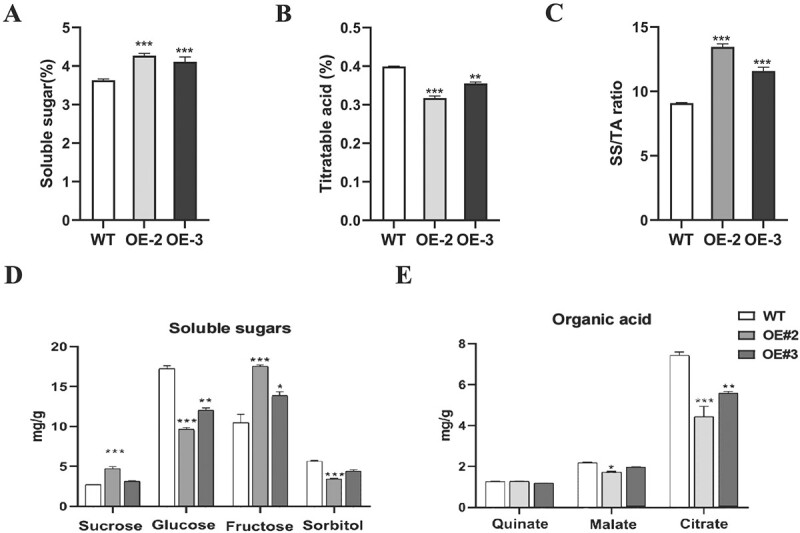
Overexpression of *PpbZIP44* significantly affected the contents of soluble sugars and organic acids in tomato fruits. A. SS contents. B. TA contents. C. SS/TA ratio. D. Individual soluble sugar content. E. Individual organic acid content. The results are averages ± SE (n ≥ 3) of WT and transgenic lines, each with three technical replicates. The asterisks indicated values that were determined by the Dunnett *t* test to be significantly different from the WT control (^*^P < 0.05, ^**^P < 0.01, ^***^P < 0.001).

Sugar and organic acids were further measured in transgenic tomato fruits. The total soluble sugar content (SS) was increased significantly, whereas the titratable acid (TA) content was decreased significantly in both two transgenic tomato lines compared with WT fruits, as shown in Fig. 4A and 4B. The sugar: acid ratio in OE-2 and OE-3 was 13.45 and 11.57, respectively, which was much higher than that in WT (9.10) (Fig. 4C). We further examined the content of soluble sugars and organic acids using high performance liquid chromatography (HPLC). Fructose was significantly induced in *E8::PpbZIP44* fruits. However, sorbitol was significantly reduced. There were no significant changes in sucrose content in OE-3 (Fig. 4D)*.* Malate and citrate contents were significantly reduced (Fig. 4E).

**Table 1:** Amino acid composition in *PpbZIP44* transient transformed pear fruits

**Amino acids**	**Empty vector**	**OE-bZIP44**	**RNAi-bZIP44**
Asp	244.15 ± 10.13	275.94 ± 13.08	187.62 ± 14.53^**^
Thr	144.87 ± 7.13	193.82 ± 5.49^*^	170.18 ± 29.43
Phe	178.20 ± 2.96	209.33 ± 4.96^***^	148.10 ± 3.70^***^
Met	109.87 ± 4.76	198.45 ± 3.46^***^	96.64 ± 1.94^**^
Glu	54.71 ± 0.65	55.29 ± 3.32	44.50 ± 1.46^**^
Ala	42.17 ± 2.80	46.03 ± 1.99	38.04 ± 2.80
Cys	51.30 ± 6.72	45.99 ± 3.80	54.87 ± 1.05
Val	54.46 ± 5.44	44.60 ± 0.07^*^	55.39 ± 1.98
Ser	39.35 ± 2.11	34.13 ± 2.13^*^	43.11 ± 1.46
Tyr	27.50 ± 1.10	47.10 ± 1.35^***^	17.94 ± 2.04^***^
Ile	24.10 ± 2.62	25.00 ± 1.73	23.59 ± 0.08
Pro	34.08 ± 2.60	12.70 ± 0.36^***^	39.87 ± 1.52^*^
NH3	7.22 ± 0.51	8.79 ± 0.46	6.39 ± 1.00
Total	1011.98	1279.35	926.24

Note: Amino acid levels were given as μg·g^−1^ fresh weight of pear fruits (±SE). All measurements were performed in triplicate. Asterisks in the same row indicate significant differences relative to empty control (^*^P < 0.05, ^**^P < 0.01, ^***^P < 0.001).

The amino acid biosynthesis and metabolism in transgenic and WT tomato fruits were significantly different. Tyramine and nicotianamine (NA) had significantly higher contents in transgenic tomato fruits. Hcy, Met, Glu, Gsh, Phe, and Val were increased ~1.45- to 1.7-fold in the *E8::PpbZIP44* tomato fruits. Tyr and Asp were also slightly induced in corresponding *E8::PpbZIP44* tomato fruits. On the other hand, Pro level was 2.3-fold lower in the transgenic tomato fruits (Supplementary Table S5).

The contents of most unsaturated fatty acids were increased by 1- to 3-fold in the *E8:PpbZIP44* fruits, compared with WT (Table 2). For examples, punicic acid (9Z,11E,13Z-octadecatrienoic acid), 9S-hydroxy-10E,12Z-octadecadienoic acid, and 9-hydroxy-13-oxo-10-octadecenoic acid were significantly increased >2-fold in the *E8:PpbZIP44* fruits. In addition, some other unsaturated fatty acids such as linoleoylglycerol, crepenynic acid, linolenic acid, (9Z,11E)-octadecadienoic acid, and eicosadienoic acid were also increased in *E8:PpbZIP44* fruits. However, 2-Dodecenedioic acid was slightly decreased in the fruits of E8:PpbZIP44 than that in the fruit of WT (Table 2).

**Table 2:** Differentially expressed unsaturated fatty acids in *E8::PpbZIP44* versus WT fruits

**Compounds**	**VIP**	**Log2FC**	**Type**
1-α-Linolenoyl-glycerol	1.37	1.37	up
2-Linoleoylglycerol	1.02	1.14	up
1-Linoleoylglycerol	1.14	1.47	up
2-Dodecenedioic acid	1.44	−1.84	down
Crepenynic acid	1.51	1.16	up
γ-Linolenic acid	1.49	1.13	up
α-Linolenic acid	1.52	1.16	up
Punicic acid (9Z,11E,13Z-octadecatrienoic acid)	1.38	2.60	up
Linoleic acid	1.26	1.14	up
(9Z,11E)-Octadecadienoic acid	1.26	1.29	up
Elaidic Acid	1.28	1.17	up
11-Octadecanoic acid(Vaccenic acid)	1.31	1.32	up
9S-Hydroxy-10E,12Z-octadecadienoic acid	1.38	2.68	up
Eicosadienoic acid	1.63	1.99	up
9-Hydroxy-13-oxo-10-octadecenoic acid	1.39	2.82	up

Chalcone and flavonoid compounds were much more abundant in the fruits of *E8:PpbZIP44* than WT, with increase of 1- to 12-fold changes. 3,4,2′,4′,6’-Pentahydroxychalcone-4’-O-glucoside, isorhamnetin-3-O-(6″-acetylglucoside), homoeriodictyol, and cinnamic acid were increased >10-fold in *E8::PpbZIP44* versus WT. The overexpression of *PpbZIP44* also strongly increased other dihydro flavones such as hesperetin-7-O-(6″-malonyl) glucoside, poncirin, eucalyptin, naringin, neohesperidin, hesperidin, naringenin, and butin; flavonols such as kaempferol-3-O-(2″-o-acetyl) glucuronide, rutin, quercetin-3-o-robinobioside, 6-hydroxykaempferol-7-O-glucoside, brassicin, isohyperoside, and hyperin; isoflavones like genistin and calycosin-7-O-glucoside, and flavones like chrysoeriol-7-O-rutinoside. However, delphinidin-3-O-glucoside (Mirtillin) and Isorhamnetin-3,7-O-diglucoside were significantly decreased in the fruits of *E8:PpbZIP44* than in the fruit of WT (Table 3).

**Table 3:** Differentially expressed flavonoid compounds in *E8::PpbZIP44* versus WT

**Class**	**Metabolites**	**VIP**	**Log2FC**	**Type**
Chalcones	3,4,2′,4′,6’-Pentahydroxychalcone-4’-O-glucoside	1.62	12.88	Up
	Okanin	1.12	1.41	Up
Dihydroflavone	Homoeriodictyol	1.59	10.05	Up
	Hesperetin-7-O-(6″-malonyl)glucoside	1.54	3.35	Up
	Poncirin (Isosakuranetin-7-O-neohesperidoside)	1.23	1.93	Up
	Eucalyptin	1.49	1.86	Up
	Naringin	1.4	1.64	Up
	Neohesperidin	1.3	1.49	Up
	Hesperidin	1.03	1.39	Up
	Naringenin	1.23	1.23	Up
	Butin	1.25	1.19	Up
	6-C-Glucosyl-2-Hydroxynaringenin	1.23	1.02	Up
Flavonols	Isorhamnetin-3-O-(6″-acetylglucoside)	1.66	10.73	Up
	Kaempferol-3-O-(2”-O-acetyl)glucuronide	1.44	2.5	Up
	Rutin	1.31	1.9	Up
	Quercetin-3-O-robinobioside	1.28	1.8	Up
	6-Hydroxykaempferol-7-O-glucoside	1.27	1.72	Up
	Brassicin	1.04	1.63	Up
	Isohyperoside	1.26	1.61	Up
	Hyperin	1.36	1.47	Up
	Isorhamnetin-3,7-O-diglucoside	1.23	−3.79	Down
Isoflavones	Genistin	1.51	1.28	Up
	Calycosin-7-O-glucoside	1.46	1.24	Up
Flavones	Chrysoeriol-7-O-rutinoside	1.33	1.94	Up

### Transcriptome changes induced by *PpbZIP44* in transgenic tomato fruits

**Figure 5:**
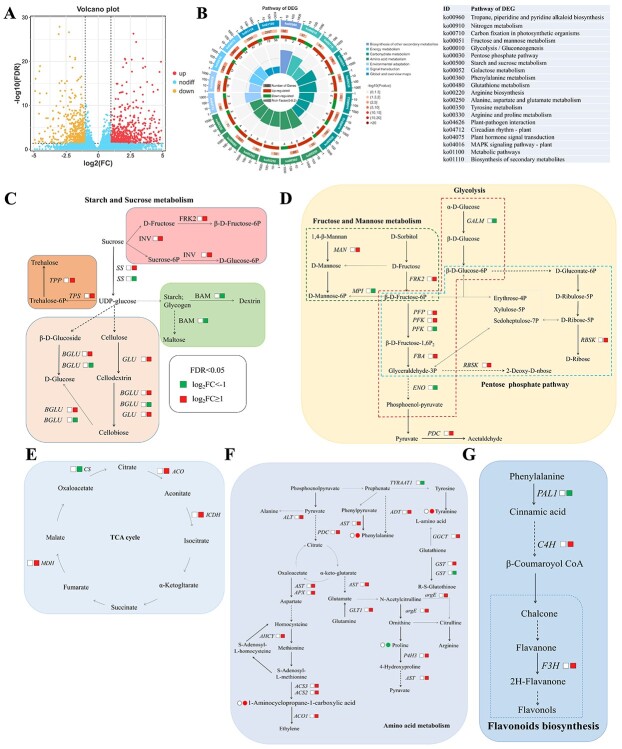
*PpbZIP44* significantly changed the primary and secondary metabolism pathway in tomato fruits based on transcriptome data. A. Volcano plot of DEGs in *E8::PpbZIP44* vs. WT fruits. B. Enriched top 20 KEGG pathway of DEGs in fruits of *E8:PpbZIP44* (OE-2) compared with WT. Detailed data are shown in the Support information Supplemental Table S6. C–E. Regulation mechanism analysis of *PpbZIP44* in the carbohydrate metabolism. DEGs induced by *PpbZIP44* in the starch and sucrose metabolism (C), fructose and mannose metabolism, glycolysis, and pentose phosphate pathway (D) and the TCA cycle (E). F. DEGs induced by *PpbZIP44* in the amino acid biosynthesis. G. DEGs induced by *PpbZIP44* in flavonoids biosynthesis. Squares representative genes. Red or green means significantly upregulated or downregulated DEGs in *E8::PpbZIP44* versus WT (white squares). *INV* encodes β-fructofuranosidase; *GLU* encodes endoglucanase; *BAM* encodes *β*-amylase; *FRK2* encodes fructokinase; *MAN* encodes mannan endo-1,4-beta-mannosidase; *MPI* encodes mannose-6-phosphate isomerase; *PFP* encodes diphosphate-dependent phosphofructokinase; *PFK* encodes 6-phosphofructokinase; *FBA* encodes fructose-bisphosphate aldolase; *RBSK* encodes ribokinase; *ADT* encodes arogenate dehydratase; *pheA* encodes prephenate dehydratase; *APX* encodes L-ascorbate peroxidase; *ALT* encodes alanine transaminase; *argE* encodes acetylomithine deacetylase; *GST* encodes glutathione S-transferase; *argE* encodes acetylornithine deacetylase; *P4H3* encodes prolyl 4-hydroxylase; *AST* encodes aspartate aminotransferase.

To elucidate how *PpbZIP44* regulates the expression of downstream genes leading to the biochemical alterations described previously, we performed transcriptomic analyses using the *E8::PpbZIP44–2* transgenic tomato line. The results showed that there were 1314 differential expression genes (DEGs) (false discovery rate (FDR) < 0.05 and |Log_2_FC| > 1) when comparing the *E8::PpbZIP44–2* versus WT tomato fruits (Fig. 5A). The top overrepresented KEGG pathways among the DEGs included carbohydrate metabolism, amino acid metabolism, and biosynthesis of secondary metabolism (Fig. 5B). The carbohydrate metabolism pathways included fructose and mannose metabolism, glycolysis, pentose phosphate pathway, starch and sucrose metabolism, and galactose metabolism (Fig. 5B and Supplemental table S6). Amino acids metabolism included glutathione metabolism, alanine, aspartate and glutamate metabolism, tyrosine metabolism, arginine and proline metabolism, and arginine biosynthesis (Fig. 5B and Supplemental Table S6). Most of the DEGs were upregulated in the carbohydrate and amino acid metabolism pathways (Fig. 5B).

DEGs in the carbohydrate metabolism pathway were further analyzed. In starch and sucrose metabolism, the expression of an *INV*, two *SS* genes, *TPS* and *TPP*, two *BGLU* genes, and *GLU* was significantly induced by the overexpression of *PpbZIP44*. On the contrary, the expression of *BAM*, linking in starch and maltose and dextrin, was significantly downregulated by *PpbZIP44* (Fig. 5C and Supplemental Table S6).

In the fructose and mannose metabolism pathway, the expression of *FRK2* and *MAN* was significantly induced by *PpbZIP44*. However, *MPI* isomerase was significantly downregulated (Fig. 5D and Supplemental Table S6). In the glycolysis and pentose phosphate pathway (PPP) pathway, the expressions of *PFP*, *PFK*, *FBA*, *PDC*, and *RBSK* were significantly upregulated in the *E8::PpbZIP44–2* transgenic tomato fruit, as shown in Fig. 5D. However, the expression of *ENO* was downregulated. In the TCA cycle, the expression of *CS* was repressed but the expression of *ACO*, *ICDH*, *and MDH* was induced in the transgenic tomato fruits (Fig. 5E).

In the amino acid biosynthesis pathway, 18 DEGs were found in *E8:PpbZIP44* tomato fruits versus WT fruits. Most of those, including *ALT, AST, ADT, APX, PAL, GLT1, argE, ADH2, HPD, GCCT, three GSTs, GDH, AHCY,* and *P4H3*, were significantly upregulated (Fig. 5F and supplementary Table S6). Flavonoids biosynthesis-related DEGs, such as *C4H* and *F3H*, were substantially upregulated by the overexpression of *PpbZIP44* (Fig. 5G).

### The expression changes of genes induced by *PpbZIP44* in transformed pear fruits

**Figure 6:**
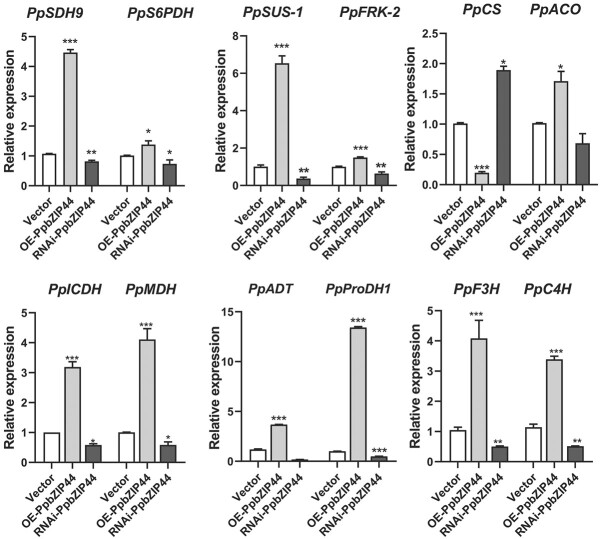
qRT-PCR analysis of gene expression related to sugar, amino acid, and flavonoid metabolism pathways and the TCA cycle in transiently transgenic pear fruits. Samples were collected in transformed pear fruits at 7 days after injecting overexpression (OE- *PpbZIP44*) or RNAi (RNAi- *PpbZIP44*) or empty vector (Vector) constructs*.* The results are average ± SE of three biological replicates, each with three technical replicates. The asterisks indicated values that were determined by the Dunnett *t* test to differ from their empty vector or WT control significantly (^*^P < 0.05, ^**^P < 0.01, ^***^P < 0.001).

To further examine and verify the changes in the expression levels of DEGs identified from the transcriptome analyses in the tomato system, we measured the transcript abundances of 12 DEGs using qRT-PCR in the transient overexpression or silencing *PpbZIP44* pear fruit. The results confirmed that *PpSDH9* gene was significantly induced in OE-bZIP44 but inhibited in RNAi-bZIP44 pear fruits. The expression levels of *PpSUS*, *PpFRK*, *PpACO*, *PpICDH*, and *PpMDH* were significantly increased in OE-bZIP44. However, the expression level of *PpCS* was significantly reduced in OE-bZIP44. *PpProDH1* transcript accumulated approximately 13-fold in OE-bZIP44. In contrast, it reduced to 0.5-fold in RNAi-bZIP44. *PpADT* transcript accumulated 2-fold in OE-bZIP44, reducing to 0.45-fold in RNAi-bZIP44. The transcript abundance of *PpC4H* and *PpF3H* was strongly induced in OE-bZIP44, whereas reduced in RNAi-bZIP44 (Fig. 6).

### *PpbZIP44* directly binds to the promoters of *PpSDH9, PpADT, PpProDH1*, and *PpF3H*

**Figure 7:**
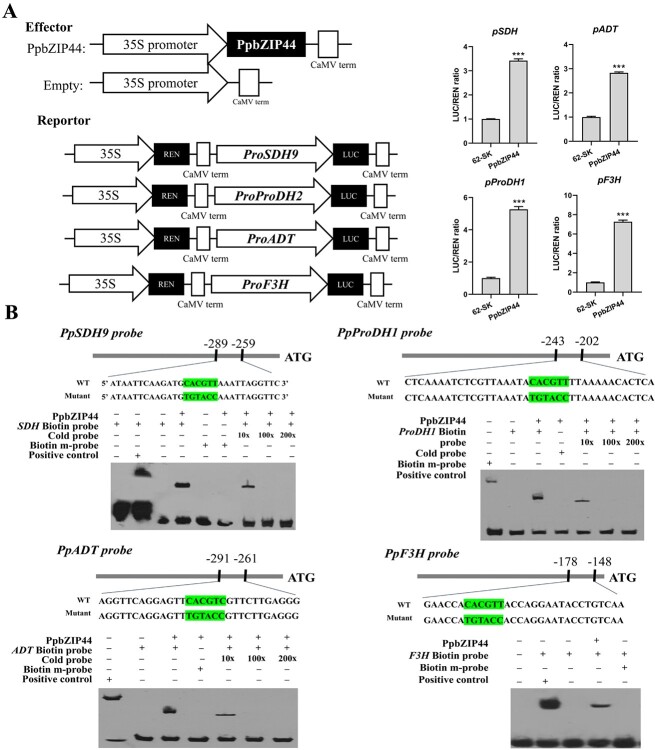
PpbZIP44 activated *PpSDH9*, *PpProDH1*, *PpADT*, and *PpF3H* by binding their promoters. A. Analysis of luciferase activity with *pSDH9*, *pProDH1*, *pADT*, and *pF3H*. 1.0-kb sequences upstream from ATG of these three genes were fused to the reporter gene Luciferase (LUC). LUC activities were assayed in transiently transformed tobacco leaves. Left: the vector construction for luciferase assays. Bars represent the mean value ± SE (n ≥ 10). The data were analyzed using the Dunnett *t* test (^***^P < 0.001). B. EMSA of PpbZIP44 binding to the *pSDH9*, *pProDH1*, *pADT*, and *pF3H* containing ACGT core site. The autoradiograph shows the DNA–protein complex of the biotin-labeled probe. Biotin probe is 30 bp of *pSDH9*, *pProDH1*, *pADT*, and *pF3H* promoter, with the CACGTT sequence; 10×, 100×, and 200× are the relative contents of the competitor probe to the detector probe. Biotin m-probe is 30 bp of *pSDH9*, *pProDH1*, *pADT*, and *pF3H* with the CACGTT mutation as TGTACC sequence.

To understand how bZIP44 regulates the expression of the downstream target genes, we performed the promoter analysis for identifying the G-boxes *cis*-element and the dual luciferase transient expression assays. We identified G-boxes *cis*-element in the 2000-bp promoter of these DEGs by *PpbZIP44* (Supplementary Table S7). Among the promoters of the 72 upregulated genes induced by *PpbZIP44*, 21 contained G-box motifs. Next, we identified typical G-boxes in the promoter of these DEGs that are involved in sorbitol and fructose metabolism, amino acids biosynthesis, and flavonoids biosynthesis, finding the promoters of *PpSDH9*, *PpADT, PpProDH1,* and *PpF3H* with G-boxes *cis*-elements (Supplementary Table S7). The regulation of *PpbZIP44* on these genes was hypothesized*.* The promoter sequences (within 1 kb) of *PpSDH9, PpADT, PpProDH1*, and *PpF3H* from ‘Sucui 1’ pear fruit were cloned and analyzed using PlantCARE (Supplemental Table S8). Three to six G-box *cis*-acting elements (CACGTT), which were predicted to recruit S1-bZIP, were found in the promoter of these four genes. The 1.0-kb fragments were cloned into the pGreen 0800-luciferase (LUC) vector for dual luciferase transient expression assays. Compared with the control level, the coexpression of *35S::PpbZIP44* and *pSDH9::LUC, pProDH1::LUC, pADT::LUC*, or *pF3H::LUC* resulted in a 3.42-, 2.82-, 5.46-, and 7.27-fold increase in LUC activity, separately (Fig. 7A). To further verify the binding of PpbZIP44 to the G-box in the promoter of these genes, we performed an electrophoretic mobility shift assay (EMSA). A 30-bp fragment (position −289 to −259 of *pSDH9*, −291 to −261 of *pADT*, −243 to −202 of *pProDH1*, and −178 to −148 of *pF3H*) was used as a probe for the EMSA (Fig. 7B). Explicit binding of the PpbZIP44 protein to the biotin-labeled target probes was detected in the polyacrylamide gel. In contrast, signals were undetectable for the protein–mutant probe complex with nucleotide changed from CACGTT to TGTACC. The signals were reduced or faded away by adding cold probes (Fig. 7B). These results suggested that *PpbZIP44* was directly bound to the *cis*-element of the *PpSDH9, PpADT, PpProDH1*, and *PpF3H* promoters and induced their transcript, enhancing the accumulation of fructose, phenylalanine, and flavonoids but deleting proline.

## Discussion

### *PpbZIP44* modulates carbohydrate metabolism of fruit

Soluble sugar accumulation determines fruit sweetness and nutritional quality at harvest. Therefore, increasing sweetness is an important breeding objective and a vital driver of consumer preference in many species [23]. Fructose is the predominant and the sweetest monosaccharide in the fruit of most pear cultivars [24, 25]. The content of fructose directly determines the quality of fresh pears. Changes in sorbitol metabolism substantially affect fructose content. In a recent study, overexpression of apple *MdABI3* increased fructose content by inducing *MdSDH2* expression [7]. Pear *SDH* genes were identified, and their expression patterns were analyzed [26]. However, it is unclear how regulating sorbitol metabolism contributes to the change in fructose content in pear fruit. Here, we found that pear *PpbZIP44* was involved in regulating the expression of a sorbitol–fructose convert. During fruit development and ripening processes in pear, *PpbZIP44* displayed significantly higher transcript abundance at stage 5, in which cell division and expansion occurs and the most significant transcriptional changes and fruit grow and metabolites associated with quality traits are accumulated [27]. Most DEGs involved in the primary metabolism were upregulated in this stage [27]. Our results suggest that *PpbZIP44* is a positive regulator of soluble sugars accumulation in pear fruits. Transiently overexpressed and gene-silenced pear fruits with *PpbZIP44* ORF confirmed the role of *PpbZIP44* in promoting *PpSDH9* expression and enhancing PpSDH enzyme activity, fructose and sucrose accumulation, and reducing sorbitol accumulation.

*SDH* catalyzes the reversible sorbitol–fructose reactions and is found in core eudicot species and monocots [28]. Compatible with the role in the initial metabolic steps of carbohydrate metabolism, *SDHs* are essential for the normal growth of plants and stress response [28–31]. Unlike pear and other Rosaceous trees, *Zea mays* does not transport sorbitol through its phloem, but instead synthesizes sorbitols in the kernel itself [32]. In maize, *Sdh1* is essential during early kernel development and in synthesizing sorbitol from fructose [33]. Conversely, in this study,the upregulation of *PpSDH9* by overexpression of *PpbZIP44* demonstrated its central role in fructose accumulation from sorbitol in pear fruits, which is consistent with the role of *MdSDH2* in apple fruit [7]. *SDH* catalyzes the conversion of fructose to sorbitol or sorbitol to fructose in a reversible manner, which might depend on species such as usual sorbitol producers or non-usual sorbitol ones [31]. In apple fruit, a large amount of sorbitol is converted into fructose and 80% of the total carbon flux goes through fructose [7], entering glycolysis and PPP. In this work, overexpression of *PpbZIP44* substantially upregulated the expression of *SUS*, *FRK2*, *PFP*, *PFK*, and *FBA* in the glycolytic direction*.* G-boxes *cis*-elements were found in the promoter of these genes, implying the bound of PpbZIP44 to these genes (Supplementary Table S7). Accordingly, our study provides evidence supporting the notion that *PpbZIP*44 is involved in soluble sugars accumulation and guides carbon flux to the downstream pathway from fructose.

Interestingly, down- or up-regulation of *SDH* genes, as well as *S6PDH* and *A6PR*, modulates sorbitol contents, leading to stamen development, pollen tube growth, and other stress responses [34–37]. SnRK1 promoted sorbitol metabolism by activating SDH enzyme and phosphorylating PpSDH protein in peach [38]. Most recently, overexpression of *MdSnRK1* increased the transcript abundance of *MdSDH1* and *MdA6PR* through SnRK1-mediated phosphorylation of MdbZIP39 [39]. Whether bZIP39 and bZIP44 interact with each other and whether or how SnRK1 mediates this interaction require further study in the future. Increased *SDH* expression and SDH activities allowed a higher carbon flux through sorbitol–fructose metabolism for plant growth and development with enough sorbitol supply [39]. The various roles of *PpbZIP*44-mediated *PpSDH9* expression on the growth and development and stress response of pear still need to be investigated.

Organic acids are significant factors determining the fruit sugar:acid ratio. Engineering organic acid metabolism substantially improved fruit traits [3]. In pear fruit, organic acids were mainly composed of quinate, citrate, and malate. Quinate can be synthesized from either 3-dehydroquinate or shikimate; these two reactions are catalyzed by QDH and QD, respectively [40]. Here, there were no significant differences in the expression level of *QDH* and *QD* and other genes in the shikimate pathway between *E8::PpbZIP44* and WT. No significant differences were observed in the content of quinate either in the overexpressed *PpbZIP44* pear or tomato fruits compared with WT. Citrate and malate control fruit acidity and are synthesized in the mitochondrion but accumulate in the vacuole [41, 42]. Citrate biosynthesis and conversion mediated by *CS* and *ACO*, or malate catalysis mediated by *MDH*, is responsible for a change in citrate or malate contents [42]. The influx and efflux and the capacity of other parts of the TCA cycle affect the import or export rate of citrate and malate [42]. In previous research, there were no apparent effects of manipulating a range of enzymes that have been proposed to control fruit citrate or malate accumulation on increasing carboxylic acids in fruits [42]. However, manipulating transcription factors such as MYB, bHLH, WRKY, and ERF family members regulated citrate or malate level [43, 44]. In Arabidopsis, induced *AtbZIP11* depleted the levels of intermediates in the TCA cycle [13]. Here, fruit-specific promoter driving overexpression of *PpbZIP44* led to significant enrichment of the TCA cycle in transgenic tomato fruits with decreasing content of α-Ketoglutaric acid, citrate, and malate. Transient expression of *PpbZIP44* significantly decreased the level of citrate and malate in OE transgenic pear fruits, which substantially contributed to the higher sugar:acid ratio in transgenic fruits.

### *PpbZIP44* regulated amino acid biosynthesis and mediated carbon flux toward secondary metabolism and flavonoid accumulation

Amino acids are pivotal in human nutrition as a source of nutraceutical compounds or essential dietary components [10]. The genes in the amino acid metabolism pathway are involved in plant response to abiotic stress [45]. The *ProDH* gene encodes a proline dehydrogenase and catalyzes the catabolism of proline [12]. In Arabidopsis, AtbZIP11 can bind to the promoters of *ProDH* and *ASN1* to induce their expression [12]. Here, 13 more folds increase of *PpProDH1* expression was induced by *PpbZIP44*. *PpbZIP44* induced rapid proline catabolism and significant enrichment of PPP, glycolysis, and the TCA cycle. We speculated that the accelerated proline cycle was coupled to the PPP, driving sugar phosphates and glycolysis for downstream anabolic pathways. Intermediates in the glycolysis pathway and the TCA cycle provide substrates and energy for the biosynthesis of amino acids and fatty acids [10, 46]. Amino acid biosynthesis pathways were significantly enriched and a series of genes involved in the pathways were upregulated. Significantly changes in Asp, Glu, Thr, Met, Val, and Ser contents illustrated the positive role of *PpbZIP44* in prompting the accumulation of these amino acids. In addition, *PpbZIP44* induced significant enrichment of ‘pantothenate and CoA biosynthesis’, ‘linoleic acid metabolism’, ‘alpha-Linolenic acid metabolism’, and ‘biosynthesis of unsaturated fatty acids’ and significantly increased unsaturated fatty acids contents. All together suggested that manipulating carbohydrate metabolism and accelerating proline catalysis and the TCA cycle through *PpbZIP44* are new and efficient strategies not only in sugar accumulation but also adjusting the balance between sugar, organic acids, and other primary metabolites and eventually affecting fruit quality, as well as role in balancing carbon to nitrogen metabolism in Arabidopsis [13].

The critical role of this TF on secondary metabolism was further determined by metabolism and transcriptome in transgenic tomato fruits and transient transgenic pear fruits. Phenylalanine is biosynthesized through the shikimate pathway and arogenate pathway [47]. *ADT* encodes arogenate dehydratase and catalyzes the final step of the arogenate pathway. Moderate expression of *ADT* increased flux through the phenylalanine biosynthesis [47]. Interestingly, other TFs such as *PgMyb308-like* can regulate the expression of genes in the shikimate pathway and promote the accumulation of shikimate, aromatic amino acids, and total lignins but reduce the contents of multiple flavonoids [48]. However, in this study, overexpression of *PpbZIP44* induced the expression of *ADT* and the increase of phenylalanine levels in fruits rather than activating the differential expression of genes in the shikimate pathway. Phenylalanine provides the substrates for these secondary metabolites, including flavonoids. Dihydro flavone contents displayed a remarkable increase in transgenic fruits. Dual-luciferase assays and EMSA demonstrated that *PpbZIP44* strongly activates *PpF3H* promoters and induces its expression. Notably, the content of many phenolic acids and the expression of genes in the lignin pathway, cutin, suberin and wax biosynthesis and zeatin biosynthesis were significantly downregulated, which was firstly found in the present study and need to further illustrate the molecular mechanism in the future.

Although the metabolic diversity of fruits in different species exists, e.g. soluble sugars were the major metabolites in pear fruits, whereas of considerably lower levels in tomato fruits. Large amounts of alkaloids were detected in tomato fruits but not in pear fruits. Total metabolic pathways and signatures related to quality formation, such as sugars, acids, flavonoids, phenylpropanoids, lipids, and amino acids, are highly conserved among fruits [6]. Due to the difficulty of obtaining stable transgenic pear fruits in fruit quality-related research, a stable transformation system in tomato fruits could be a supplemental function analysis of a transient transformation system. Fruit-specific promoter drivers powerful TFs, which might be involved in considerable growth and development processes, successfully avoiding the adverse effect on plant growth. RNA-Seq in *E8::PpbZIP44* tomato fruits and qRT-PCR examination in transient transformation pear fruits provide plenitude shreds of evidence for the role of *PpbZIP44* in fruit multimetabolism reprogramming and quality formation.

In summary, we generated a model to elucidate the regulation mechanism of *PpbZIP44* on substantial changes in multiquality-related compounds. *PpbZIP44* could bind to the *PpSDH9* promoter to induce its expression. The enhanced activity of SDH promoted the conversion of sorbitol to fructose, resulting in a considerable accumulation of fructose. *PpbZIP44* substantially induced the higher expression of *SUS*, *FRK2*, *PFP*, *PFK*, and *FBA* in the glycolytic direction*. PpbZIP44* recruited the *PpProDH1* promoter to accelerate proline catabolism, which might couple PPP to drive sugar phosphates and glycolysis for downstream anabolic pathways. Depleting the intermediate of the TCA cycle facilitated the biosynthesis of amino acids and affected the sugar:acid ratio. Moreover, *PpbZIP44* bound to the *PpADT* promoter to induce their expression, leading to metabolic flux toward phenylalanine. *PpbZIP44* bound to the *PpF3H* promoter and induced the increase of dihydro flavone contents. Metabolic flux toward dihydro flavone might decrease lignin formation, which is involved in cell stone formation and crucial for the perception of the quality of pear fruit. Regarding Asian pears, high sugar content, moderate acidity, crispy, few stone cells, and high nutritional value are desirable traits. Identification and functional characterization of *PpbZIP44* provides a novel strategy for molecular breeding through metabolic engineering for fruit quality improvement.

## Materials and methods

### Plant materials

Fruits at different development stages in ‘Sucui 1’ were collected every 15 days after full bloom (DAFB) until 90 DAFB for stage 6. Samples at 93 DAFB and 100 DAFB were collected for stages 7 and 8 from the Pear Germplasms Resources at the Institute of Pomology, Jiangsu Academy of Agricultural Sciences, Nanjing, Jiangsu, China. The trees were cultivated as follows: 3.0 m × 5.0 m spacing, with rows oriented in the east–west direction. Trees were pruned into an arch shape. Base fertilizer was applied in the autumn and the trees were irrigated twice weekly. Alfalfa in winter and white clover in summer between rows were cut up regularly and left on the ground. The pear fruit peel (0.3 cm) was removed and sarcocarp samples were frozen in liquid nitrogen. The sarcocarps were collected with three independent biological replicates for qRT-PCR, transient transformation, or DNA extraction.

### Identification of S1-bZIP proteins in the sand pear

All bZIP protein sequences of Arabidopsis were obtained from Arabidopsis Information Resource (http://arabidopsis.org/; release 10.0). Pear genome sequences were obtained from National Genomics Data Center (https://bigd.big.ac.cn/gwh.). The Hidden Markov Model profile of bZIP_1 (PF00170) and bZIP_2 (PF07716) was obtained from the Pfam database (http://pfam.sanger.ac.uk) [49] and SMART database (http://smart.embl-heidelberg.de) [50]. A local BLAST search was completed with Arabidopsis bZIP proteins as queries to remove redundancies by checking all identical sequences manually. All the confirmed pear bZIP proteins were aligned using MUSCLE (version 3.8.31) [51]. A phylogenetic tree was generated by analyzing the multiple sequence alignment with the Clustal W [51], and the alignment results were displayed using Jalview [52]. The sand pear S1-bZIP proteins were further identified through a local BLAST search of Arabidopsis, tomato, tobacco, grape, apple, strawberry, rice, cucumber, banana, and petunia protein sequences, downloaded from Solanaceae Genome Annotation Database (https://solgenomics.net) and Plant Transcription Factor Database (http://planttfdb.cbi.pku.edu.cn; release 3.0).

### Transient overexpression and RNAi silencing of genes in pear fruits

The transient transformation and expression analysis were performed as Yao described [53]. Briefly, total RNA was extracted from ‘Sucui 1’ sarcocarps and used as the template to synthesize cDNA. The mORF sequence of *PpbZIP44* (EVM0042575) was ligated to the pGreenII 62-SK or pSAK277 vectors using OE- or RNAi-primers (Table S1) under the control of the 35S promoter [53]. *Agrobacterium tumefaciens*-mediated transient transformation was performed in ‘Sucui 1’ pear fruits at 90 DAFB [53]. Fruits transformed with pGreen II 62-SK or pSAK277 empty vector were used as controls. The fruits of three biological replicates, including five fruits at least for each experiment, were collected for sugars, organic acids, amino acids, and enzyme activity measurements, and a qRT-PCR examination after 7 days.

### Fruit-specific driving vector construction and tomato transformation

The CaMV 35S promoter was eliminated and E8 promoter (GenBank: DQ317599) was amplified (Table S1) and linked to pBI121-GFP [18]. The fragment was then ligated into pBI121-S1-bZIP-GFP vectors. The cloned inserts, including the E8 promoter and target genes, were confirmed by sequencing. Explants were prepared and transformation was performed as described by Sagor *et al* [18]. The regenerated plantlets were then transferred to the rooting medium until the shoot grows to 2- to 4-cm long. The DNA of tomato plantlets was extracted and analyzed by RT-PCR with the bZIP44-gF2 and GFP-FCX_R. Positive transgenic plantlets were confirmed by RT-PCR and sequenced, after which they were planted in soil in a greenhouse.

### Widely targeted metabolomics analysis based on UPLC-MS/MS system

Compounds were extracted and examined using A UPLC (SHIMADZU Nexera X2) and Tandem mass spectrometry MS/MS (Applied Biosystems 4500 QTRAP) as previously described [22, 26]. With the methods we used, all metabolites can be measured simultaneously using widely targeted metabolomics based on MRM [22, 26]. The relative signal intensities of metabolites were normalized by dividing them according to the intensities of the internal standard (lidocaine, 0.1 mg·l^−1^) first and after that log_2_ transform them to get further normalization for normality improvement [54]. Then we calculated the area of each peak to quantify the metabolites. The PCA and orthogonal partial least squares discriminant analysis (OPLS-DA) analysis were completed to assess the differences in compounds. VIP ≥ 1 and fold-change ≥2 or ≤ 0.5 were used as threshold levels for selecting differentially abundant metabolites.

### RNA isolation, sequencing and differential gene expression analyses

Each biological replicate consisted of at least five fruits from different trees. Total RNA from leaves and fruits was extracted from the sarcocarp or tomato flesh with Trizol (Invitrogen, USA) and the Ambion RiboPure™ Kit (Ambion, USA) [55]. Then, mRNA was prepared using Oligo (DT) system. cDNA libraries were constructed and sequenced with nova-seq6000 platform based on the manufacturer’s instructions. Clean reads of the nine samples were pooled and mapped to the tomato genome (Sol Genomics Network, *S. lycopersicum* 3.0) with HISAT2 (HISAT2 (daehwankimlab.github.io)) [56]. Transcripts were assembled and quantified with StringTie [57]. Differential analysis was performed with FDR < 0.05 and |log2FC| > 1 as described [58]. Additionally, GOseq (adjusted *P* < 0.05) was used for the GO term enrichment analysis [59], whereas KO (KEGG Ortholog database) was used to enrich DEGs to different pathways [60].

### Soluble sugar and organic acid content measurement

The content of the total SS and TA of fruits was measured according to the previous description [17]. Sucrose, glucose, fructose, sorbitol, quinate, malate, and citrate levels in pear and tomato fruits were measured using HPLC [17]. Briefly, 0.8 g of frozen pear sarcocarps were ground into powder and then resuspended with 800 μl of extraction buffer containing 0.2% metaphosphoric acid and 80% ethanol. The mixture was treated with an ultrasonicator for 10 min, incubated overnight at 4°C, and centrifuged at 10 000 rpm for 10 min at 25°C. 800 μl of supernatant was concentrated for 3 h at 30°C, dissolved in 1.6 ml deionized water (Milli-Q water), and then filtered through 0.22-μm Sep-Park filter paper. Organic acids and soluble sugars were analyzed with an HPLC system. We calculated the area of each peak to quantify the metabolites according to an external standard calibration method.

### Amino acid content measurement

For amino acid content, pear fruit samples were directly frozen and ground in liquid nitrogen after transient transformation for 7 days. Total amino acids were extracted with a mixed 100- to 200-mg sample and 750 μl of 80% ethanol in an ultrasonic bath at 50°C for 30 min. Then we centrifuged samples, collected the supernatants and removed the solvent by freeze-drying. The extract was added with 5.7 M of hydrochloric acid, hydrolyzed overnight under a closed condition at 110°C, dissolved in water, dried again, and resuspended in 1 ml of sodium citrate buffer (pH 2.2). The composition of amino acid in all samples was tested by Hitachi amino acid automatic analyzer LA8080.

### SDH enzyme activity measurement

Activities of SDH were measured using the commercial kits (catalog #:ab252902) based on the instruction. Quantifying enzyme activities were measured through a spectrophotometer with UV-VISO 2450 at 25°C (Shimadzu, Kyoto, Japan).

### qRT-PCR

cDNA was synthesized using the HiScript III RT SuperMix Kit with gDNA wiper (Vazyme, China) for a qRT-PCR analysis with specific primers (Table S1). The pear *GAPDH* and tomato *SlACTIN* genes were used as internal standards for data normalization. Average threshold cycle values were counted based on three independent biological replicates and then calculated the relative transcript level of each gene [61].

### Dual luciferase assay

The dual luciferase reporter assay was performed as previously described [7]. The coding sequence of full-length PpbZIP11 (EVM0042575) was cloned into the pGreenII62-SK to produce the effector construct. A 1000-bp promoter sequence (named *pSDH9, pProDH1, pADT* and *pF3H*) of *PpSDH9, PpProDH1, PpADT*, and *PpF3H* (Supplemental Table S8) was introduced separately into the pGreenII0800-LUC vector. The transformed *A. tumefaciens*-strain GV3101 cells were used to coinoculate the young leaves of *Nicotiana benthamiana* tobacco plants. The LUC and renilla luciferase (REN) activities were measured with a dual luciferase assay system (Promega, USA) and an Infinite M200 (TECAN, Switzerland), recorded as a ratio of LUC to REN.

### EMSA

The EMSA assay was conducted as previously described [62]. Briefly, the *PpbZIP44* ORF was ligated into the pET-28a prokaryotic expression vector. The resulting plasmid was inserted into *Escherichia coli* Rosetta cells and cultured on LB medium with 40 mg·l^−1^ kanamycin to overexpress the PpbZIP44 protein via the addition of isopropyl β-D-1-thiogalactopyranoside and an overnight incubation at 16°C. The recombinant protein was purified with the HisTrap HP column (GE Healthcare, Piscataway, NJ, USA). The biotin-labeled DNA fragments (*pSDH*9: ATAATTCAAGATGCACGTTAAATTAGGTTC**;***pProDH1*: CTCAAAATCTCGT-. TAAATACACGTTTTAAAAACACTCA; *pADT*: AGGTTCAGGAGTTCACGTCGTTCTT-. GAGGG; *pF3H*: GAACCACACGTTACCAGGAATACCTGTCAA) were synthesized, annealed, and used as WT probes, whereas the corresponding site-directed (CACGTT) mutated DNA fragments (TGTACC) were used as mutant probes. WT probes without label served as the competitors. The interaction between PpbZIP44 and the probes and the binding signals were detected with the LightShift EMSA Optimization and Control Kit and the Chemiluminescent Nucleic Acid Detection Module Kit (Thermo Fisher, MA, USA).

### Statistical analysis

Dunnett *t* test in the multiple comparison analysis of variance was applied to determine whether any of the differences between the mean values for treatments and genotypes were significant.

## Acknowledgments

We thank Prof. Songling Bai for kindly providing the genome database of ‘Cuiguan’ pear. We thank Chuan-Bei Jiang (Genepioneer Biotechnologies, Nanjing 210014, China) for providing bioinformatics analysis. This work was supported by Jiangsu Agricultural Science and Technology Independent Innovation Fund [Grant No. CX(22)3010], the General Program of National Natural Science Foundation of China (Grant No. 31872078, 32272669), Jiangsu Modern Agricultural Industry Technology System (Grant No. JATS[2022]437, JATS[2022]438), Jiangsu Agricultural Key New Varieties Innovation (Grant No. PZCZ201726), Jiangsu Province Seed Industry Revitalization Unveiled Project: Breeding of New Pear Cultivars with High-quality and Extreme Early Ripening (Grant No. JBGS[2021]084). The funders had no role in study design, data collection, and analysis.

## Author Contributions

H.W., L.J., and C.Z.J. designed and supervised the research. W.J., B.L.S., and Y.H.C. suggested the research. H.W. and K.X.X. wrote the manuscript. B.B.U. and C.Z.J. revised the manuscript. W.H. conducted the RNA-seq library and metabolites preparation, processed the RNA-seq data and metabolites data, gene clone, transformation, HPLC, EMSA, dual luciferase assay, and statistics. K.X.X. performed RT-qPCR and physiology experiments. H.W. and G.F.Y. performed transient transformation. Y.D.W. performed sequences analysis. Q.S.Y. and X.G.L. prepared pear fruits. All authors read and approved the final manuscript.

## Data availability

All the RNA-seq data are available and have been deposited in the National Center for Biotechnology Information Sequence Reads Archive (PRJNA947778). Other relevant data are presented within the paper and its supplementary files.

## Conflict of Interest

The authors declare that they have no conflict of interest.

## Supplementary data

Supplementary data is available at *Horticulture Research* online.

## Supplementary Material

Web_Material_uhad140

## References

[1] Adaskaveg JA , Blanco-Ulate B. Targeting ripening regulators to develop fruit with high quality and extended shelf life. Curr Opin Biotechnol. 2023;79:102872 36621222 10.1016/j.copbio.2022.102872

[2] Ruan YL . Sucrose metabolism: gateway to diverse carbon use and sugar signaling. Annu Rev Plant Biol. 2014;65:33–67 24579990 10.1146/annurev-arplant-050213-040251

[3] Batista-Silva W , Nascimento VL, Medeiros DB et al. Modifications in organic acid profiles during fruit development and ripening: correlation or causation? Front Plant Sci. 2018;9:1689 30524461 10.3389/fpls.2018.01689 PMC6256983

[4] Fei X , Hu H, Luo Y et al. Widely targeted metabolomic profiling combined with transcriptome analysis provides new insights into amino acid biosynthesis in green and red pepper fruits. Food Res Int. 2022;160:111718 36076459 10.1016/j.foodres.2022.111718

[5] Sun H , Zhou X, Zhou Q et al. Disorder of membrane metabolism induced membrane instability plays important role in pericarp browning of refrigerated 'Nanguo' pears. Food Chem. 2020;320:126684 32229394 10.1016/j.foodchem.2020.126684

[6] Pott DM , Osorio S, Vallarino JG. From central to specialized metabolism: an overview of some secondary compounds derived from the primary metabolism for their role in conferring nutritional and organoleptic characteristics to fruit. Front Plant Sci. 2019;10:835 31316537 10.3389/fpls.2019.00835 PMC6609884

[7] Wang Z , Ma B, Yang N et al. Variation in the promoter of the sorbitol dehydrogenase gene *MdSDH2* affects binding of the transcription factor MdABI3 and alters fructose content in apple fruit. Plant J. 2022;109:1183–98 34888978 10.1111/tpj.15624

[8] Tanase K , Shiratake K, Mori H et al. Changes in the phosphorylation state of sucrose synthase during development of Japanese pear fruit. Physiol Plant. 2002;114:21–6 11982930 10.1046/j.0031-9317.2001.10137.x

[9] Tanase K , Shiratake K, Yamaki S. The mechanisms of sucrose accumulation in Japanese pear (*Pyrus pyrifolia* Nakai) fruit. Acta Hortic. 2002;479-487:479–87

[10] Trovato M , Funck D, Forlani G et al. Editorial: amino acids in plants: regulation and functions in development and stress defense. Front Plant Sci. 2021;12:772810 34733310 10.3389/fpls.2021.772810 PMC8559698

[11] Carmona-Gutierrez D , Zimmermann A, Kainz K et al. The flavonoid 4,4′-dimethoxychalcone promotes autophagy-dependent longevity across species. Nat Commun. 2019;10:651 30783116 10.1038/s41467-019-08555-w PMC6381180

[12] Hanson J , Hanssen M, Wiese A et al. The sucrose regulated transcription factor bZIP11 affects amino acid metabolism by regulating the expression of *ASPARAGINE SYNTHETASE1* and *PROLINE DEHYDROGENASE2*. Plant J. 2008;53:935–49 18088315 10.1111/j.1365-313X.2007.03385.x

[13] Ma J , Hanssen M, Lundgren K et al. The sucrose-regulated *Arabidopsis* transcription factor bZIP11 reprograms metabolism and regulates trehalose metabolism. New Phytol. 2011;191:733–45 21534971 10.1111/j.1469-8137.2011.03735.x

[14] Garg A , Kirchler T, Fillinger S et al. Targeted manipulation of bZIP53 DNA-binding properties influences *Arabidopsis* metabolism and growth. J Exp Bot. 2019;70:5659–71 31257431 10.1093/jxb/erz309 PMC6812703

[15] Wang H , Zhang Y, Norris A et al. S1-bZIP transcription factors play important roles in the regulation of fruit quality and stress response. Front Plant Sci. 2022;12:802802 35095974 10.3389/fpls.2021.802802 PMC8795868

[16] Rook F , Gerrits N, Kortstee A et al. Sucrose-specific signaling represses translation of the *Arabidopsis ATB2* bZIP transcription factor gene. Plant J. 1998;15:253–63 9721683 10.1046/j.1365-313x.1998.00205.x

[17] Xing S , Chen K, Zhu H et al. Fine-tuning sugar content in strawberry. Genome Biol. 2020;21:230 32883370 10.1186/s13059-020-02146-5 PMC7470447

[18] Zhang Y , Li S, Chen Y et al. Heterologous overexpression of strawberry *bZIP11* induces sugar accumulation and inhibits plant growth of tomato. Sci Hortic. 2022;292:110634

[19] Sagor GH , Berberich T, Tanaka S et al. A novel strategy to produce sweeter tomato fruits with high sugar contents by fruit-specific expression of a single bZIP transcription factor gene. Plant Biotechnol J. 2016;14:1116–26 26402509 10.1111/pbi.12480 PMC11388862

[20] Konarska A . The relationship between the morphology and structure and the quality of fruits of two pear cultivars (*Pyrus communis* L.) during their development and maturation. Sci World J. 2013;2013:1–13 10.1155/2013/846796 PMC3845256 24327806

[21] Gao Y , Yang Q, Yan X et al. High-quality genome assembly of 'Cuiguan' pear (*Pyrus pyrifolia*) as a reference genome for identifying regulatory genes and epigenetic modifications responsible for bud dormancy. Hortic Res. 2021;8:197 34465760 10.1038/s41438-021-00632-w PMC8408243

[22] Chen W , Gong L, Guo Z et al. A novel integrated method for large-scale detection, identification, and quantification of widely targeted metabolites: application in the study of rice metabolomics. Mol Plant. 2013;6:1769–80 23702596 10.1093/mp/sst080

[23] Jaeger SR , Andani Z, Wakeling IN et al. Consumer preferences for fresh and aged apples: a cross-cultural comparison. Food Qual Prefer. 1998;9:355–66

[24] Doty TE . Fructose sweetness: a new dimension. Cereal Foods World. 1976;21:62–3

[25] Nishio S , Hayashi T, Shirasawa K et al. Genome-wide association study of individual sugar content in fruit of Japanese pear (*Pyrus* spp.). BMC Plant Biol. 2021;21:378 34399685 10.1186/s12870-021-03130-2 PMC8369641

[26] Dai M , Shi Z, Xu C. Genome-wide analysis of sorbitol dehydrogenase (SDH) genes and their differential expression in two sand pear (*Pyrus pyrifolia*) fruits. Int J Mol Sci. 2015;16:13065–83 26068235 10.3390/ijms160613065 PMC4490486

[27] Xu J , Yan J, Li W et al. Integrative analyses of widely targeted metabolic profiling and transcriptome data reveals molecular insight into metabolomic variations during apple (*Malus domestica*) fruit development and ripening. Int J Mol Sci. 2020;21:4797 32645908 10.3390/ijms21134797 PMC7370097

[28] Jia Y , Wong DC, Sweetman C et al. New insights into the evolutionary history of plant sorbitol dehydrogenase. BMC Plant Biol. 2015;15:101 25879735 10.1186/s12870-015-0478-5 PMC4404067

[29] Shi XP , Ren JJ, Yu Q et al. Overexpression of *SDH* confers tolerance to salt and osmotic stress, but decreases ABA sensitivity in *Arabidopsis*. Plant Biol (Stuttg). 2018;20:327–37 29125673 10.1111/plb.12664

[30] Liu X , Fan HM, Liu DH et al. Transcriptome and metabolome analyses provide insights into the watercore disorder on "Akibae" pear fruit. Int J Mol Sci. 2021;22:4911 34066340 10.3390/ijms22094911 PMC8124519

[31] Pleyerová I , Hamet J, Konradova H et al. Versatile roles of sorbitol in higher plants: luxury resource, effective defender or something else? Planta. 2022;256:13 35713726 10.1007/s00425-022-03925-z

[32] Zimmermann MH , Ziegler H. List of sugars and sugar alcohols in sieve-tube exudates. In: Zimmermann MH, Milburn JA (eds.), Transport in Plants I: Phloem Transport. Springer-Verlag: Berlin, 1975,158–92

[33] de Sousa SM , Paniago MG, Arruda P et al. Sugar levels modulate sorbitol dehydrogenase expression in maize. Plant Mol Biol. 2008;68:203–13 18566893 10.1007/s11103-008-9362-0

[34] Deguchi M , Bennett AB, Yamaki S et al. An engineered sorbitol cycle alters sugar composition, not growth, in transformed tobacco. Plant Cell Environ. 2006;29:1980–8 16930323 10.1111/j.1365-3040.2006.01573.x

[35] Meng D , Li C, Park HJ et al. Sorbitol modulates resistance to *Alternaria alternata* by regulating the expression of an *NLR* resistance gene in apple. Plant Cell. 2018;30:1562–81 29871985 10.1105/tpc.18.00231 PMC6096587

[36] Meng D , He M, Bai Y et al. Decreased sorbitol synthesis leads to abnormal stamen development and reduced pollen tube growth via an MYB transcription factor, *MdMYB39L*, in apple (*Malus domestica*). New Phytol. 2018;217:641–56 29027668 10.1111/nph.14824

[37] He X , Meng H, Wang H et al. Quantitative proteomic sequencing of *F_1_* hybrid populations reveals the function of sorbitol in apple resistance to *Botryosphaeria dothidea*. Hortic Res. 2022;9:uhac115 35937862 10.1093/hr/uhac115 PMC9346975

[38] Yu W , Peng F, Wang W et al. SnRK1 phosphorylation of SDH positively regulates sorbitol metabolism and promotes sugar accumulation in peach fruit. Tree Physiol. 2021;41:1077–86 33576402 10.1093/treephys/tpaa163 PMC8190949

[39] Meng D , Cao H, Yang Q et al. SnRK1 kinase-mediated phosphorylation of transcription factor bZIP39 regulates sorbitol metabolism in apple. Plant Physiol. 2023;192:2123–42 37067900 10.1093/plphys/kiad226 PMC10315300

[40] Guo J , Carrington Y, Alber A et al. Molecular characterization of quinate and shikimate metabolism in *Populus trichocarpa*. J Biol Chem. 2014;289:23846–58 24942735 10.1074/jbc.M114.558536 PMC4156088

[41] Etienne A , Génard M, Lobit P et al. What controls fleshy fruit acidity? A review of malate and citrate accumulation in fruit cells. J Exp Bot. 2013;64:1451–69 23408829 10.1093/jxb/ert035

[42] Morgan MJ , Osorio S, Gehl B et al. Metabolic engineering of tomato fruit organic acid content guided by biochemical analysis of an introgression line. Plant Physiol. 2013;161:397–407 23166354 10.1104/pp.112.209619 PMC3532270

[43] Huang XY , Wang CK, Zhao YW et al. Mechanisms and regulation of organic acid accumulation in plant vacuoles. Hortic Res. 2021;8:227 34697291 10.1038/s41438-021-00702-z PMC8546024

[44] Zhang L , Ma B, Wang C et al. MdWRKY126 modulates malate accumulation in apple fruit by regulating cytosolic malate dehydrogenase (*MdMDH5*). Plant Physiol. 2022;188:2059–72 35078249 10.1093/plphys/kiac023 PMC8968328

[45] Liu W , Wang Q, Zhang R et al. Rootstock-scion exchanging mRNAs participate in the pathways of amino acids and fatty acid metabolism in cucumber under early chilling stress. Hortic Res. 2022;9:uhac031 35184197 10.1093/hr/uhac031 PMC9039506

[46] Jiang L , Geng D, Zhi F et al. A genome-wide association study provides insights into fatty acid synthesis and metabolism in *Malus* fruits. J Exp Bot. 2022;73:7467–76 36112134 10.1093/jxb/erac372

[47] Yoo H , Shrivastava S, Lynch JH et al. Overexpression of arogenate dehydratase reveals an upstream point of metabolic control in phenylalanine biosynthesis. Plant J. 2021;108:737–51 34403557 10.1111/tpj.15467

[48] Dhakarey R , Yaritz U, Tian L et al. A Myb transcription factor, *Pg*Myb308-like, enhances the level of shikimate, aromatic amino acids, and lignins, but represses the synthesis of flavonoids and hydrolyzable tannins, in pomegranate (*Punica granatum* L.). Hortic Res. 2022;9:uhac008 35147167 10.1093/hr/uhac008 PMC9113223

[49] Finn RD , Bateman A, Clements J et al. Pfam: the protein families database. Nucleic Acids Res. 2014;42:D222–30 24288371 10.1093/nar/gkt1223 PMC3965110

[50] Letunic I , Doerks T, Bork P. SMART 6: recent updates and new developments. Nucleic Acids Res. 2009;37:D229–32 18978020 10.1093/nar/gkn808 PMC2686533

[51] Chenna R , Sugawara H, Koike T et al. Multiple sequence alignment with the Clustal series of programs. Nucleic Acids Res. 2003;31:3497–500 12824352 10.1093/nar/gkg500 PMC168907

[52] Waterhouse AM , Procter JB, Martin DM et al. Jalview version 2—a multiple sequence alignment editor and analysis workbench. Bioinformatics. 2009;25:1189–91 19151095 10.1093/bioinformatics/btp033 PMC2672624

[53] Yao G , Ming M, Allan AC et al. Map-based cloning of the pear gene *MYB114* identifies an interaction with other transcription factors to coordinately regulate fruit anthocyanin biosynthesis. Plant J. 2017;92:437–51 28845529 10.1111/tpj.13666

[54] Chen W , Wang W, Peng M et al. Comparative and parallel genome-wide association studies for metabolic and agronomic traits in cereals. Nat Commun. 2016;7:12767 27698483 10.1038/ncomms12767 PMC5059443

[55] Wang H , Stier G, Lin J et al. Transcriptome changes associated with delayed flower senescence on transgenic petunia by inducing expression of *etr1-1*, a mutant ethylene receptor. PLoS One. 2013;8:e65800 23874385 10.1371/journal.pone.0065800 PMC3706537

[56] Kim D , Langmead B, Salzberg SL. HISAT: a fast spliced aligner with low memory requirements. Nat Methods. 2015;12:357–60 25751142 10.1038/nmeth.3317 PMC4655817

[57] Pertea M , Pertea GM, Antonescu CM et al. StringTie enables improved reconstruction of a transcriptome from RNA-seq reads. Nat Biotechnol. 2015;33:290–5 25690850 10.1038/nbt.3122 PMC4643835

[58] Robinson MD , McCarthy DJ, Smyth GK. edgeR: a bioconductor package for differential expression analysis of digital gene expression data. Bioinformatics. 2010;26:139–40 19910308 10.1093/bioinformatics/btp616 PMC2796818

[59] Young MD , Wakefield MJ, Smyth GK et al. Gene ontology analysis for RNA-seq: accounting for selection bias. Genome Biol. 2010;11:R14 20132535 10.1186/gb-2010-11-2-r14 PMC2872874

[60] Kanehisa M , Sato Y, Kawashima M et al. KEGG as a reference resource for gene and protein annotation. Nucleic Acids Res. 2016;44:D457–62 26476454 10.1093/nar/gkv1070 PMC4702792

[61] Wang H , Lin J, Chang Y et al. Comparative transcriptomic analysis reveals that ethylene/H2O2-mediated hypersensitive response and programmed cell death determine the compatible interaction of sand pear and Alternaria alternata. Front Plant Sci. 2017;8:195 28261248 10.3389/fpls.2017.00195 PMC5309250

[62] Rio DC . Electrophoretic mobility shift assays for RNA-protein complexes. Cold Spring Harb Protoc. 2014;2014:435–40 24692495 10.1101/pdb.prot080721

