# Supplementary material for: A pear S1-bZIP transcription factor PpbZIP44 modulates carbohydrate metabolism, amino acid, and flavonoid accumulation in fruits
Source: Hortic Res. 2023 Jul 21;10(8):uhad140. doi: 10.1093/hr/uhad140 (PMC10421730; doi:10.1093/hr/uhad140)
Supplement: Web_Material_uhad140 [file web_material_uhad140.zip › Supplementary Table S1.docx]

**Table S1 Primer sequences**

| **Primer Name** | **Sequence (5' to 3')** |
| --- | --- |
| PpbZIP44-qF1 | AAACGACTCCCAGGTAGCATT |
| PpbZIP44-qR1 | ACAACCTCGCTCATAACACCC |
| PpbZIP53-qF1 | TTGTCCAATCTTTCTCCGTCAT |
| PpbZIP53-qR1 | TCCAGGTGCTGTTGCTTCC |
| PpbZIP44_OE_F1 | actagtggatccaaagaattcATGGCTTCTTCAAGCGGAAA |
| PpbZIP44_OE_R1 | tcattaaagcaggactctagaTTAATAGTATTGGTGAAGCATGTCAGC |
| EVM0041928_OE_F1 | actagtggatccaaagaattcATGGCTTCGTCCAGTGGGA |
| EVM0041928_OE_R1 | tcattaaagcaggactctagaTCAGTAGTGAAACATATCTGTAGAGGCC |
| PpbZIP44_RNAi_F1 | actagtggatccaaagaattcGTCTTGAAAGCGCAGATGGG |
| PpbZIP44_RNAi_R1 | tcattaaagcaggactctagaATAGTATTGGTGAAGCATGTCAGCA |
| PpbZIP53_RNAi_F1 | actagtggatccaaagaattcTCTGTTCTCAGAGCCCAAGCTG |
| PpbZIP53_RNAi_R1 | tcattaaagcaggactctagaGTAGTGAAACATATCTGTAGAGGCCAT |
| bZIP44-gF2 | GCATAAACATCACCACCCAG |
| GFP-FCX_R | TTGTGGCCGTTTACGTCGCC |
| PpSDH9_qF | GTCCGTTCCACTGTATGGTT |
| PpSDH9_qR | GCAAAGGAGTGGAGGAGTC |
| Pear-GAPDH_F | TGGTGTGAACGAGAAGGAAT |
| Pear-GAPDH_R | CCCTCAACAATCCCAAACC |
| S6PDH_F | TAAGTACCAAAAAAAATACAACGGC |
| S6PDH_R | GAAATACGGAAAGAGCGTGGCA |
| FRK2_F2 | TGGCTCTTCATTCTACTG |
| FRK2_R2 | GACTGTCCGTTGACTAAA |
| SUS1_F1 | CAGGCATGAAAGAAGCAAGGTC |
| SUS1_R1 | GTAGCAGAAATGCCATCCACTG |
| CS_F | GTCTGTGGTAGATGAAGTTGGAGAG |
| CS_R | CTAGTTCAGTAAGTATGGGAGGCAC |
| ACO_F | TTGTTGGTTTGTCGGTCTTTCG |
| ACO_R | TATCACATTGGTCGTTTATCGT |
| MDH_F | GGAGATGCTGGTGTTGTTGAATGC |
| MDH_R | CCTTCTGGATGCTTGCTGCTAACT |
| ICDH_F | AAACATCCCTCGCCTTATCC |
| ICDH_R | CCCCTCCCCTGTAAAGTCAT |
| SuDH-F2 | CCATTGCTTCGGCGTCGTCTA |
| SuDH-R2 | ACTCCTTGAGGTCGATCTCGTACT |
| *C4H*-F | TCACGTCCACGTAACGTTGTG |
| *C4H*-R | TGATACGTCTCATTTTTCTCCAATG |
| ADT-F | ATCTCACAAAACCCGCCCTA |
| *ADT*-R | CTCGATTTGTCAGATGGCGG |
| ProDH_F | ACCACCATCTCTGCCATCTC |
| ProDH_R | CATGAGCCTAGAACGCATGA |
| F3H_F | GGAGAAAGACAAAGTGGAGATAAAGC |
| F3H_R | ACAAGAAGTGGAAAGGCAAAGTTAC |
| EVM0041187-3F | AGATAGAGTCTGCGAACAACG |
| EVM0041187-3R | CAAAGATATGGAGGACCGAGT |
| EVM0005411-4F | CCAGCCTAAACATCACAAGC |
| EVM0005411-4R | TGGAGCCCACCATTGTTT |
| EVM0026513-6F | CTCCTGACAAGGGATAACAACC |
| EVM0026513-6R | ACCCATCACCTCCACCAATA |
| EVM0014652-1F | AGCAGCAGCACCTGGATGAT |
| EVM0014652-1R | ACGCCGCCGTTGATGTAA |
