# Supplementary figures and images for: A pear S1-bZIP transcription factor PpbZIP44 modulates carbohydrate metabolism, amino acid, and flavonoid accumulation in fruits
Source: Hortic Res. 2023 Jul 21;10(8):uhad140. doi: 10.1093/hr/uhad140 (PMC10421730; doi:10.1093/hr/uhad140)

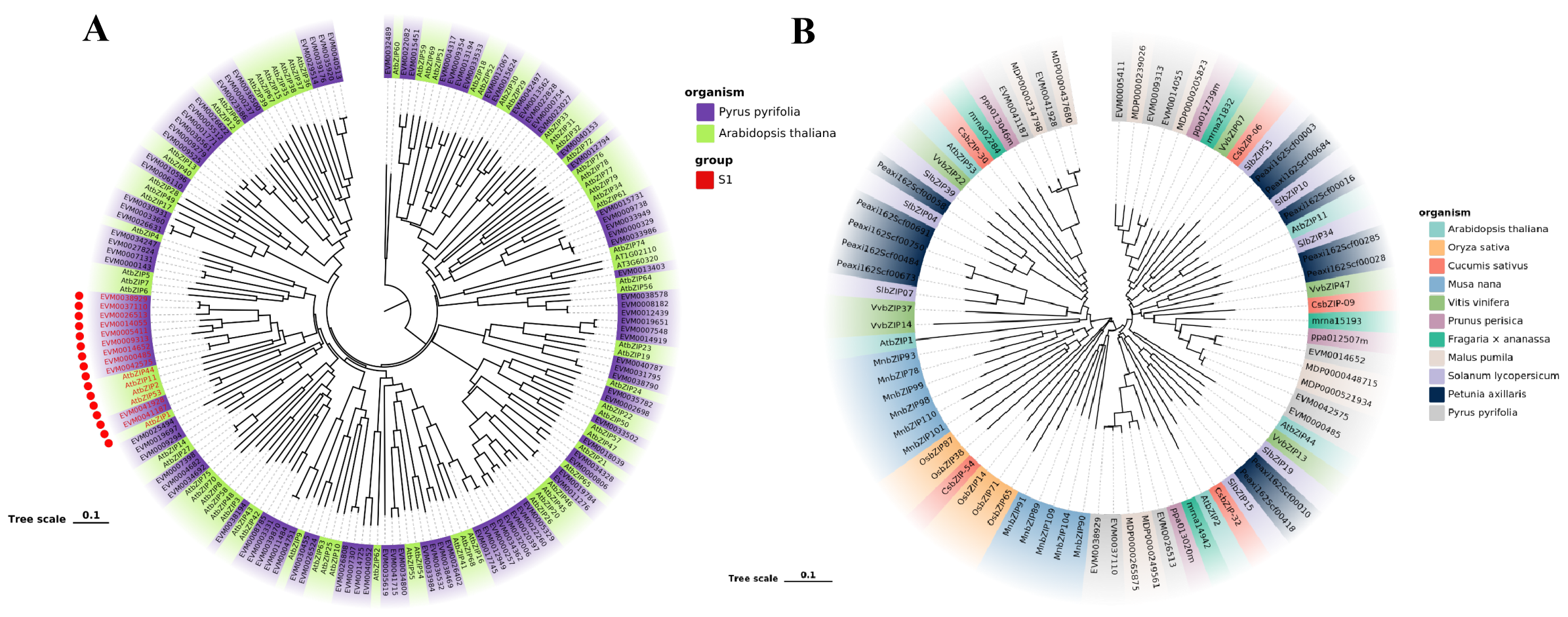

Supplement: Web_Material_uhad140 [file web_material_uhad140.zip › Supplementary Fig. S1.tif]

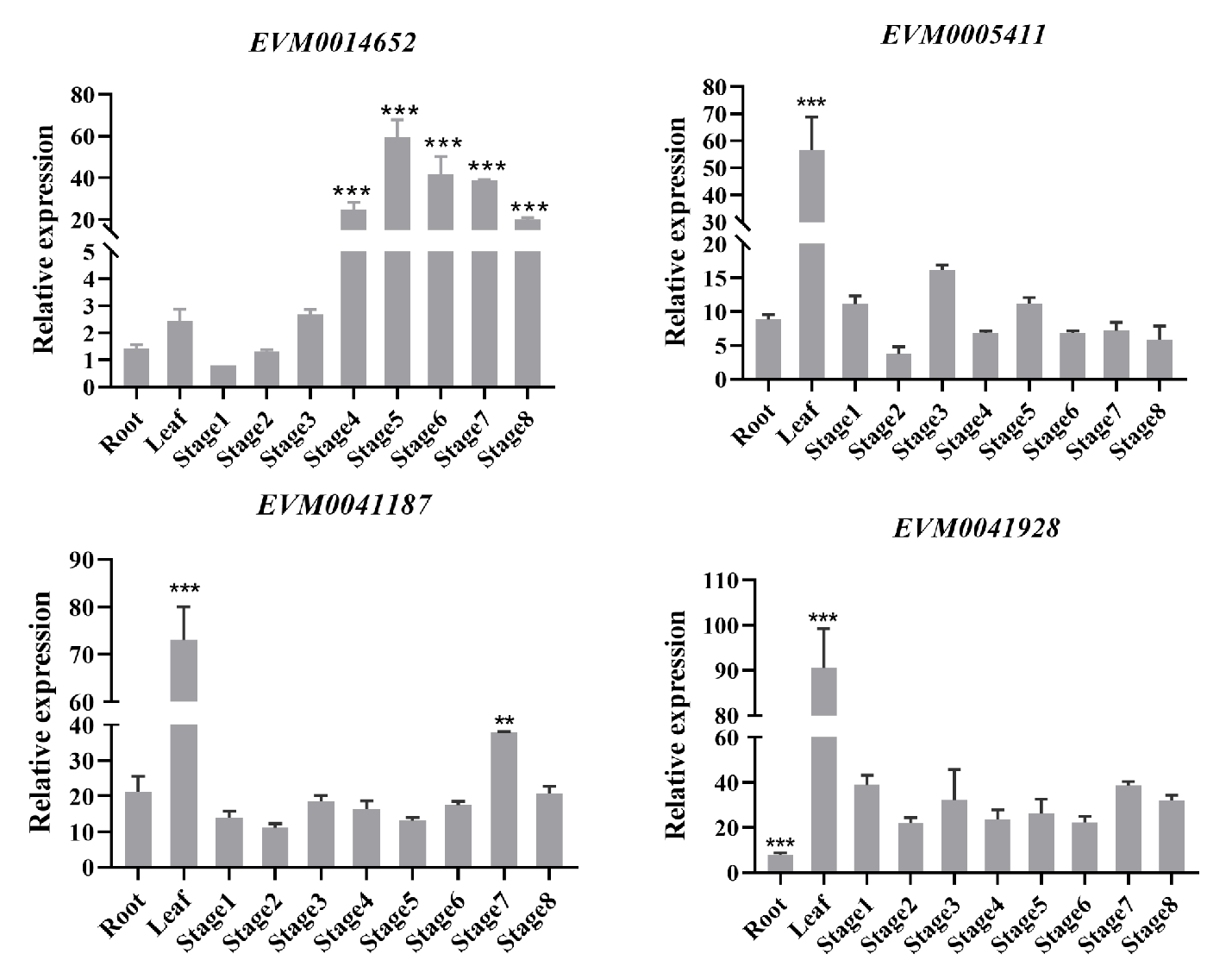

Supplement: Web_Material_uhad140 [file web_material_uhad140.zip › Supplementary Fig. S2.tif]
